# Equivocal tests after contrast stress-echocardiography compared with invasive coronary angiography or with CT angiography: CT calcium score in mildly positive tests may spare unnecessary coronary angiograms

**DOI:** 10.1186/s12947-017-0119-2

**Published:** 2018-02-06

**Authors:** Nicola Gaibazzi, Guido Pastorini, Andrea Biagi, Francesco Tafuni, Claudia Buffa, Silvia Garibaldi, Francesca Boffetti, Giorgio Benatti

**Affiliations:** Department of Cardiology, Parma University Hospital, Via Gramsci 14, 43123 Parma, Italy

**Keywords:** Stress-echocardiography, Computed tomography angiography, Coronary artery disease, Equivocal tests

## Abstract

**Background:**

Imaging stress tests are not ideally accurate to predict anatomically obstructive CAD, leading to a non-trivial rate of unnecessary iCA. This may depend on the threshold used to indicate iCA, and maybe CTA or, one step earlier, CT calcium score could spare most unnecessary iCA in only mildly positive cSE. We assessed the diagnostic accuracy of contrast stress-echocardiography (cSE) in comparison with invasive coronary angiography (iCA), and CT angiography (CTA) only in case of equivocal tests, to find hints helping reduce falsely positive cSE in the suspicion of coronary artery disease (CAD).

**Methods:**

Patients who were indicated cSE for suspected CAD between 2012 and 2016, who also underwent iCA were selected and diagnostic results compared. A second group, specifically with equivocal cSE who underwent CTA was also analyzed.

**Results:**

137 subjects with equivocal cSE and CTA and 314 with cSE (any result) and iCA were selected. In the CTA-equivocal cSE group, an Agatston score < 105 and a coronary flow reserve (CFR-LAD) <1.7 had very high negative predictive value (99%, 92% respectively) to exclude obstructive CAD. The Agatston score was the most significant incremental predictor of CAD beyond clinical variables (chi square 31 to 47, *p* < 0.001). In the iCA group a more-than-mild reversible wall motion abnormality (WMA) demonstrated high positive predictive value for CAD (89%), while CFR-LAD appeared less useful. More-than-mild reversible WMA was the most significant predictor of CAD beyond clinical variables (chi square 37.5 to 56, *p* < 0.001).

**Conclusions:**

Our data suggest iCA should be indicated only for more-than-mild reversible WMA at cSE, due to the very high positive predictive value for CAD of this finding, while mildly positive tests should be shifted to non-invasive CT, with CTA performed only for coronary calcium Agatston score > 100, since lower scores demonstrated very high negative predictive value for CAD, not justifying proceeding to CTA and even less to iCA.

## Background

Recent stress-echocardiography studies have been mostly focused on prognostic stratification [1]; however, the diagnostic gatekeeping role to invasive tests more directly impacts on patients clinical management, aiming to avoid financial and biological downstream costs of unnecessary invasive coronary angiograms (iCA). The recent NICE guidelines [2] suggest the “anatomical” multislice CT coronary angiography (CTA) approach to be preferred over functional stress-testing in the suspicion of coronary artery disease (CAD), emphasizing the general disappointment regarding the suboptimal diagnostic performance of functional tests. In fact, functional tests still over-predict obstructive CAD in 1/3 to ½ of patients who are then indicated iCA [3, 4], and, more surprisingly, this has not improved since 1977, when Erikssen et al. first reported that coronary angiograms of patients selected for suspected CAD showed “..a ratio of 2 true positives to 1 false positive” [5]. Diagnostic tests are usually forced into a binary classification, positive or negative, and imaging functional tests for suspected CAD make no exception. This simplistic classification does not fit for all patients. In today’s low-pretest probability practice [6], negative tests represent the vast majority of stress-echocardiograms, positive tests represent a minority, and a third class, not so rare, is represented by “equivocal” tests, which is impossible to classify. In our lab we suggest iCA for patients testing positive for reversible ischemia, while CTA is proposed specifically for patients with equivocal tests, in line with existing recommendations on CTA appropriateness. A wealth of variables can be collected from our routine vasodilator contrast stress-echocardiography (cSE) protocol, comprising wall motion (WM), Doppler coronary flow reserve of the left anterior descending coronary artery (CFR-LAD), myocardial perfusion, ECG abnormalities, and anginal symptoms. We assessed diagnostic accuracy data of cSE in the iCA and CTA groups, to find hints helping modify our practice and minimize false positive cSE sent to iCA. Our lab’s routine practice to assess multiple variables and indicate iCA not only on the basis of WM, but also on myocardial perfusion, CFR-LAD data or clinical suspicion, makes WM accuracy data only partially affected by referral bias.

## Methods

### Patients

**Fig. 1:**
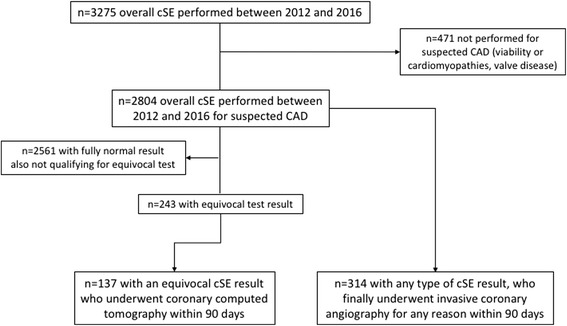
Flow diagram of patients selection in the current study. cSE = contrast stress-echocardiography, CAD = coronary artery disease

From our cSE database we retrospectively selected patients who were indicated cSE for suspected CAD between 2012 and 2016. We selected patients who were either indicated a) iCA after cSE (<90 days) for any clinical reason, whatever the cSE result, or b) multislice CTA specifically after equivocal cSE, defined as mild reversible WM abnormalities, or normal WM but abnormal findings among the other parameters assessed during cSE. Figure 1 shows the flow diagram of patients selection. Patients who underwent iCA within 90 days (either because of a positive cSE or any other clinical reason, comprising the results of other stress-tests) were selected and cSE and iCA data compared. In the second group, equivocal cSE who were indicated and underwent CTA within 90 days were also selected and results analyzed; in this CTA group we also applied the exclusion criteria of prior coronary revascularization or myocardial infarction, not applied to the iCA group, to exclude patients with possible stent-related artifacts, a known diagnostic limitation of CTA.

Diabetes mellitus was defined as a fasting plasma glucose level > 125 mg/dL or the need for insulin or oral hypoglycemic agents. Hypercholesterolemia was defined as total cholesterol >200 mg/dL or treatment with lipid lowering medications. Hypertension was defined as blood pressure > 140/90 mmHg or use of antihypertensive medication.

The study was approved by the Institutional Review Board of the Parma Medical Center and all patients gave informed consent.

### Contrast stress-echocardiography

Our protocol for accelerated high-dose dipyridamole contrast stress-echocardiography has been described elsewhere [7]; briefly, it consists in rest and peak vasodilation assessments of the following three imaging parameters: WM, myocardial perfusion and Doppler CFR-LAD for peak diastolic velocity stress/rest ratio, after small boluses of 0.5 ml SonoVue microbubble ultrasound contrast. Stress acquisition is performed after dipyridamole administration of 0.84 mg/kg infused in 6 min. Wall motion analysis is the only assessment strictly required for the protocol, perfusion imaging and CFR-LAD being assessed only when feasible, mostly depending on the specific operator preferences and technical skills. The left ventricle is divided in 17 segments according to the recommendations of the American and European Societies of Echocardiography [8]. Myocardial perfusion is visually assessed, normal myocardial perfusion after dipyridamole was assigned if myocardium was fully replenished 1.5–2 s after the end of the flash impulse, normal myocardial replenishment at rest was defined as complete replenishment within 4 s after the flash impulse. Segmental wall motion was graded as follows: normal = 1; hypokinetic = 2; akinetic = 3; and dyskinetic = 4. Reversible ischemia was defined as the occurrence of a stress-induced new dyssynergy or worsening of rest hypokinesia in ≥1 segment.

### Definition of equivocal or positive cSE test

**Fig. 2:**
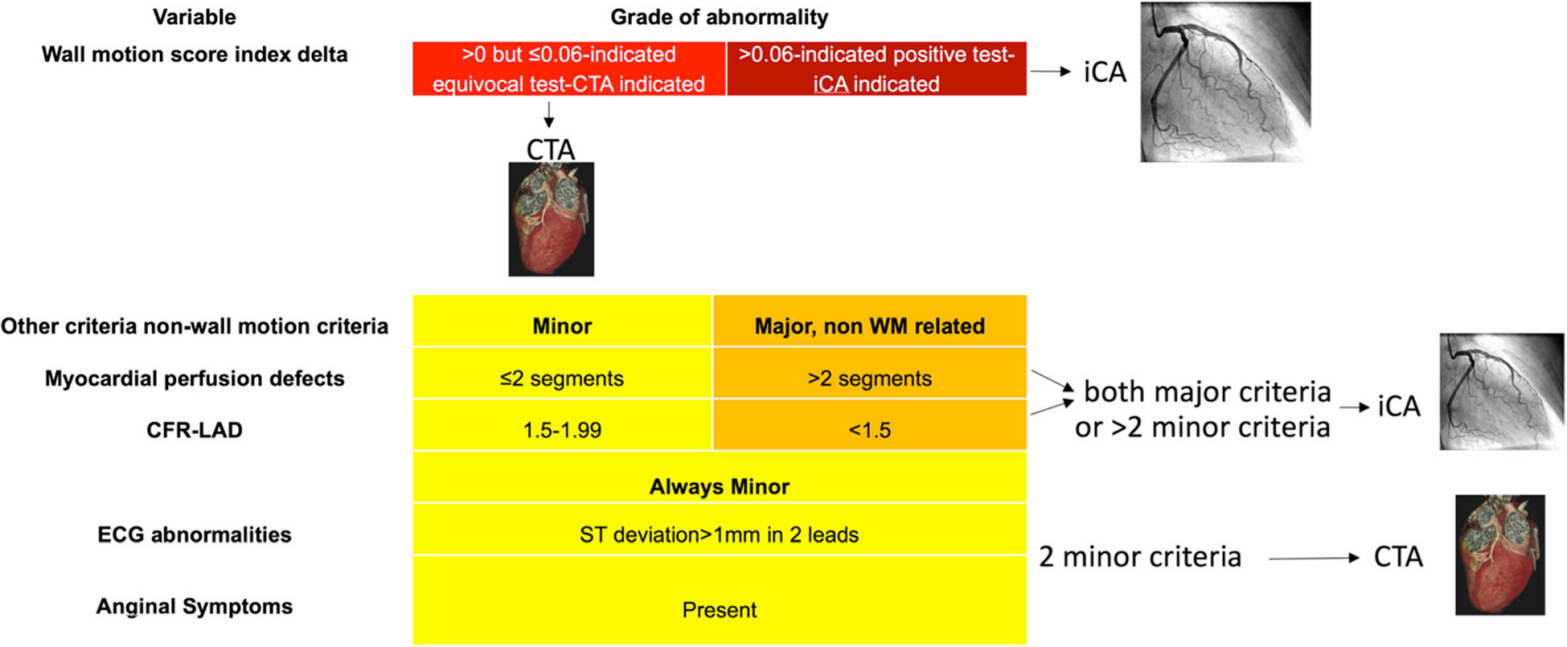
Pre-specified criteria suggested in our lab to define equivocal contrast stress-echocardiograms (who are indicated CTA) based on wall motion and the other available variables. Additionally to the standalone wall motion score index delta criterion, patients with 2 minor criteria were also defined equivocal tests and indicated CTA, while more than 2 minor or two major were also defined positive tests and indicated iCA. Yellow colour represents minor criteria, orange means major criteria, but unrelated to wall motion assessment. CTA = CT angiography, iCA = invasive coronary angiography, CFR-LAD = coronary flow reserve of the left anterior descending coronary artery. Yellow boxes identify minor criteria, Orange are major non-WM criteria, while light red and dark red indicate WM mild and more-than-mild ischemia

Following our lab guidelines, CTA was indicated only after an equivocal cSE, which was strictly defined based either on only mildly abnormal wall motion score index (WMSI) delta between rest and stress (≤0.06) as a standalone criterion or, alternatively, normal WM behavior (WMSI delta = 0) but 2 “minor” abnormal findings among the following: a) mild reversible perfusion defects (affecting ≤2 segments), b) abnormal but not extremely reduced CFR-LAD (between 1.5 and 1.99), c) reversible downsloping or horizontal ST deviation of >1 mm in at least 2 ECG leads, d) anginal symptoms at peak vasodilation. The definition of a frankly positive cSE, encompassed either a more severe WMSI delta (>0.06) as a standalone criterion or, alternatively, WMSI delta ≤0.06 but associated with >2 other ancillary abnormal variables or only 2 of such variables, but showing extremely abnormal values, limited to myocardial perfusion or CFR-LAD. Figure 2 clarifies stress-echocardiography criteria used to define equivocal cSE or positive tests in our echolab.

### Coronary Calcium and CTA

The study was performed with a Definition Flash system (Siemens, Forchheim, Germany). Gantry rotation time was 280 ms, which provides a temporal resolution of 75 ms using a heart rate independent single-segment reconstruction and high pitch up to 3.4. Detector collimation is 2 × 64 × 0.6 mm. A z-axis flying focal spot is applied which results in an acquisition of 2 × 128 slices per rotation. Two scans were performed in all patients: one to visualize coronary artery calcium and one angiography scan. First patients underwent non-enhanced prospective electrocardiography (ECG)-gated sequential scan to measure coronary calcium score. The corresponding images for calcium scoring were reconstructed with a slice width of 2.5–3 mm and slice spacing of 1.25–1.5 mm and the tube voltage was 120 kVp. A region of interest was drawn over the areas of calcification and the Agatston score was automatically calculated by the software. A cutoff value above 130 Hounsfield units defined calcification and lesion area was multiplied by a density factor derived from the maximal Hounsfield units. Scans for coronary CTA were performed with breath held in inspiration. CTA was performed with adaptive electrocardiographic pulsing. A prospective ECG-triggering high-pitch spiral mode or retrospective ECG gating spiral mode were used. All images were reviewed on a workstation (Leonardo Siemens) equipped with a dedicated software tool for calcium scoring (Calcium Scoring CT, Siemens).

### Invasive Coronary Angiography

Angiograms were performed by standard technique via radial or femoral approach. Obstructive CAD was primarily defined as stenosis >50% in any major epicardial coronary artery, but the alternative stenosis cutoff of >70% was also assessed. Left main trunk with at least 50% stenosis was always considered as >70% CAD. Invasive coronary angiography wad graded by visual inspection of the cath-lab physician performing the diagnostic procedure.

### Statistics

This was an observational retrospective study of data collected in clinical practice. All data were expressed as mean (±standard deviation) or median (and interquartile range—IQR) or number (percentage), as appropriate. Comparisons between patients with at least one significant coronary stenosis and patients with unaffected coronaries (or <50% stenosis) were made by unpaired t-test or the Wilcoxon rank sum test and test for proportions, as appropriate. We created receiver operating characteristic (ROC) curves of considered parameters, and we calculated area under the curve (AUC) to estimate their predictive power for the presence of CAD. Accuracy data were reported using common definitions of sensitivity, specificity, positive and negative predictive values. Logistic regression models were used to evaluate how demographics, clinical risk factors or imaging parameters predicted the presence of CAD and the potential incremental value using change in models chi square. Coronary artery disease was by default defined as the presence of at least one >50% visually-assessed coronary artery stenosis; when the alternative definition of more severe >70% coronary artery stenosis was used and assessed, this was expressly specified in the manuscript. A *p* value <0.05 was considered statistically significant. StatsDirect 3 statistical software (StatsDirect Ltd. http://www.statsdirect.com) was used.

## Results

Out of overall 3275 cSE performed between 2012 and 2016, we found 137 subjects satisfying the definition for equivocal cSE who underwent CTA after cSE and within 90 days, who had no history of prior myocardial infarction or revascularization (specific exclusion criteria for the CTA group), and 314 who underwent iCA within 90 days after cSE.

### Equivocal cSE and CTA data

In the 137 patients, CTA demonstrated no or <50% coronary stenosis in 96 patients (70%) (no CAD group), while in 41 (30%) demonstrated at least one >50% coronary artery stenosis (CAD group); among such 41 patients, 14 had at least one coronary artery stenosis >70% or left main disease >50%. Only 26 subjects out of the overall 41 patients in whom CTA reported at least >50% stenosis subsequently underwent iCA, which confirmed CTA findings in 22 (85%) with 4 patients (15%) being over-diagnosed by CTA with >50% stenosis, downgraded to <50% at iCA.

Baseline risk factors, cSE and CTA data are shown in Table 1, which highlights few significant differences between the CAD and no CAD groups: age was significantly higher in the group with CAD (Median 65 vs 57 y/o, *p* < 0.0001), as it was the prevalence of hypertension, active smoking and hypercholesterolemia (*p* < 0.05 for all).

**Table 1:** Baseline demographics, clinical variables, stress-echocardiography and computed tomography angiograms data in the entire population and in the subgroups with or without obstructive coronary artery disease > 50%

	Entire population(*n* = 137)	CAD > 50% (*n* = 41)	No CAD > 50% (*n* = 96)	p
Age, median (IQR)	59 (11)	65 (10)	57 (11)	<0.0001
Male Gender (%)	79 (58%) (57%)	28 (68%)	51 (53%)	0.1
Hypertension (%)	83 (60%)	30 (73%)	53 (55%)	0.048
Family history of CAD (%)	37 (27%)	11 (27%)	26 (27%)	0.9
Smoking (%)	26 (19%)	14 (34%)	12(13%)	0.003
Hypercolesterolemia (%)	62 (45%)	25 (61%)	37 (39%)	0.015
Diabetes mellitus (%)	16 (12%)	7 (17%)	9 (9%)	0.2
Coronary Calcium Agatston score, Median (IQR)	25 (175)	269 (917)	2 (42)	<0.0001
Coronary stenosis >50% but <70%	27 (20%)	27 (66%)	–	–
At least one stenosis more than 70%	14 (10%)	14 (34%)	–	–
Bridge, tortuous course or coronary anomaly, sum, (%)	21, 24, 17 = 62 (45%)	9, 6, 7 = 22 (54%)	12, 18, 10 = 40 (42%)	0.2
Any reversible WM abnormality	18 (13%)	5 (12%)	13 (13%)	0.9
Reversible myocardial perfusion abnormality	78 (56%)	21 (51%)	57 (60%)	0.37
CFR-LAD Median (IQR)	2,0 (0.2)	1,9 (0.3)	2 (0.2)	0.003

*CAD* coronary artery disease, *IQR* interquartile range, *WM* wall motion, *CFR-LAD* coronary flow reserve of the left anterior descending coronary artery

The only cSE imaging variable significantly differing between the 2 groups was CFR-LAD, which was lower in the CAD group (median 1.9 vs 2.0, *p* < 0.005), while the CT-derived coronary calcium, measured as the Agatston score, was significantly higher in the CAD group (median 269 vs 2, *p* < 0.001).

**Fig. 3:**
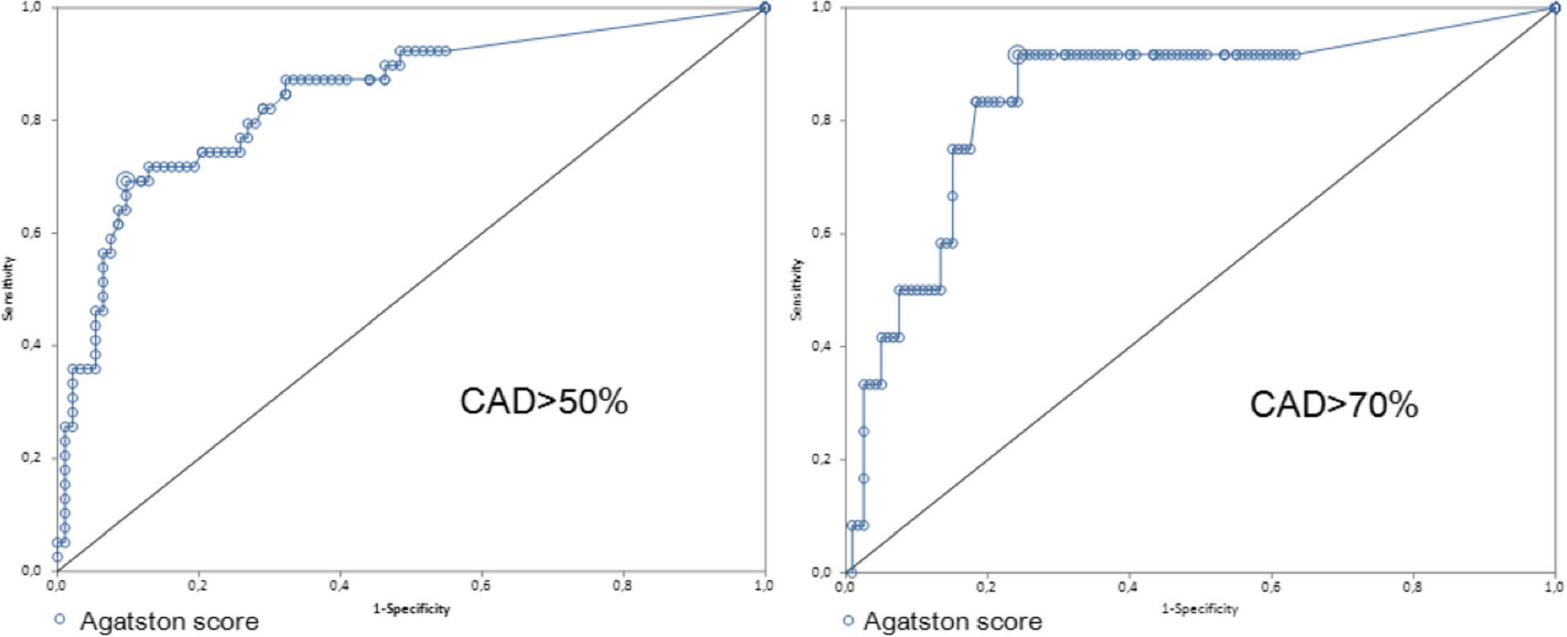
Receiver operating curves describing the accuracy data at varying Agatston score values. The best cutoff to predict CAD > 50% (left) was 150, while to predict CAD >70 (right) it was 105. CAD = coronary artery disease

Figure 3 shows the ROC curves, on the left using the calculated best cutoff of >150 for the Agatston score to predict >50% CAD at CTA, demonstrating an area under the curve (AUC) = 0.843 (95% CI 0,765 to 0,920), a negative predictive value of 87,5% (95% CI 79% to 93%) and a positive predictive value of 75% (95% CI 58% to 88%); on the right the ROC curve for the prediction of more severe >70% CAD, using the specific best cutoff of >105, showing superimposable AUC value (0.843, 95% CI 0,713 to 0,973), but an increased negative predictive value of 99% (95% CI 94% to 100%) and a decreased positive predictive value, of only 27,5% (95% CI 15% to 44%).

From the ROC curve for CFR-LAD to predict >50% CAD at CTA, the best cutoff was <1.8, demonstrating a suboptimal AUC = 0.339 (95% CI 0,234 to 0,443), a negative predictive value of 78% (95% CI 68% to 86%) and a positive predictive value of 58% (95% CI 39% to 75%), while the ROC curve recalculated for the prediction of more severe >70% CAD showed an AUC of 0.370 (95% CI 0,195 to 0,546) using the calculated best cutoff of <1.7, an increased negative predictive value of 92% (95% CI 85% to 97%) and a positive predictive value of only 28,5% (95% CI 11% to 52%).

**Fig. 4:**
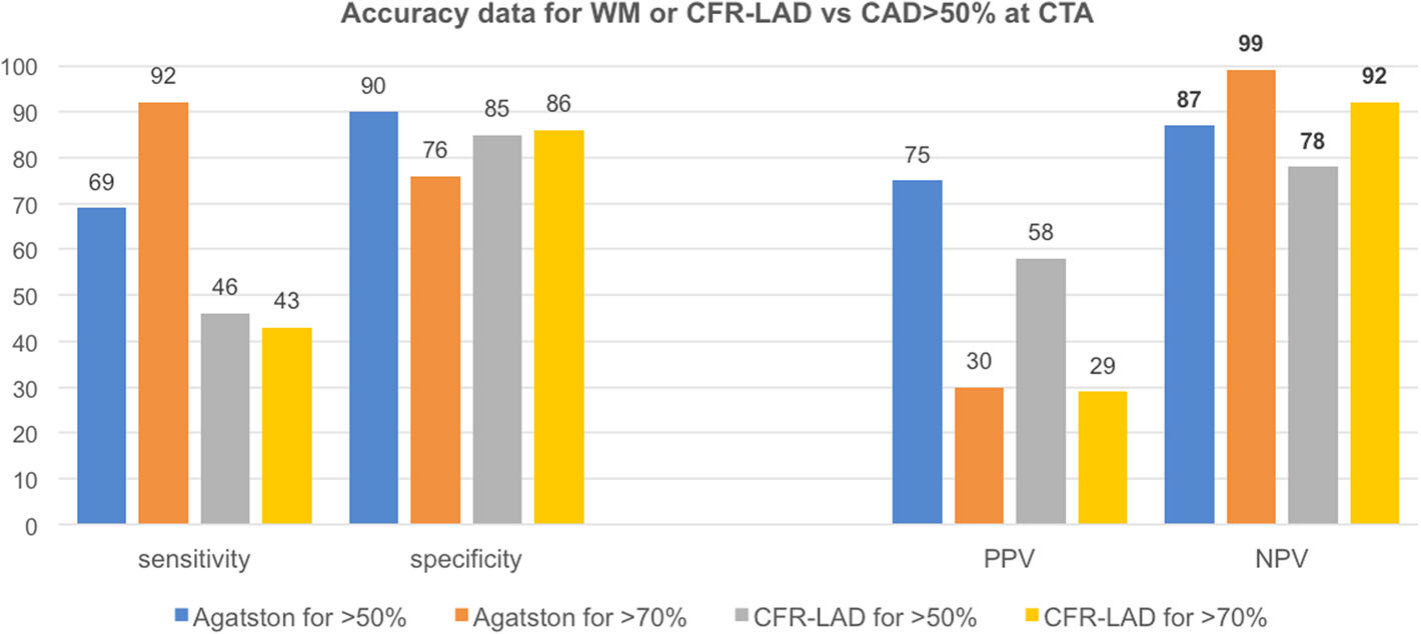
Accuracy data to predict CAD >50% and CAD >70% at CT angiography, when CFR-LAD or Agatston score were assessed as predictors. CAD = coronary artery disease, PPV = positive predictive value, NPP = negative predictive value, CFR-LAD = coronary flow reserve of the left anterior descending coronary artery

Full accuracy data in this CTA group of patients with equivocal cSE are shown in Fig. 4, highlighting the very high negative predictive value of an Agatston score < 105 (and also of a CFR < 1.7) to exclude the presence of CAD > 70%.

**Fig. 5:**
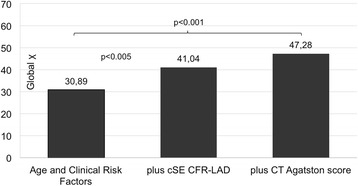
Incremental usefulness based on model chi square of adding imaging data over baseline demographics and clinical variables to predict CAD > 50%. CAD = coronary artery disease. CFR-LAD = coronary flow reserve of the left anterior descending coronary artery

Figure 5 graphically demonstrates the incremental diagnostic benefit of adding CFR-LAD data (chi square increases from 30.89 to 41.04, *p* < 0.005) or the Agatston score (chi square increases from 30.89 to 47.28, *p* < 0.001) over baseline demographics + clinical risk factors.

### Contrast Stress-echocardiography and iCA data

In the 314 patients with available iCA, 73 (23%) demonstrated no or <50% coronary stenosis in (no CAD group), while 241 (77%) demonstrated at least one >50% coronary artery stenosis (CAD group); among such 241 patients, most (*n* = 222, 92%) had also at least one coronary artery stenosis >70% or left main trunk disease >50%. Reversible wall motion abnormalities of any degree (delta WMSI > 0) were found in 206 (66%) patients, while only 164 (52%) demonstrated a reversible more-than-mild wall motion abnormality (WMSI > 0.06), highlighting that half of patients who underwent iCA did so not mainly driven by cSE wall motion results, but other cSE variables, other functional tests or clinical variables. CFR-LAD data were available only for 208 (66%) and myocardial perfusion data in 176 (56%) in this real-world cohort of 314 patients, highlighting the less than optimal feasibility of such two imaging parameters compared with standard WM when cSE data are prospectively collected in routine clinical practice, not selecting operators based on their advanced technical skills. Accuracy to predict CAD for such only partly available variables was calculated limited to patients in which they were measurable.

Baseline risk factors, cSE and iCA data are shown in Table 2, which highlights few significant differences between the CAD and no CAD groups: age was significantly higher in the group with CAD (median 67 vs 53, *p* = 0.005), as it was the prevalence of male gender, hypertension, dyslipidemia and diabetes mellitus (*p* < 0.05 for all), while smoking habit and prior myocardial infarction were apparently more frequent, but only of borderline statistical significance (*p* = 0.05).

**Table 2:** Baseline demographics and clinical variables, stress-echocardiography and invasive coronary angiograms data in the entire population and in the subgroups with or without obstructive coronary artery disease > 50%

	Entire population (*n* = 314)	CAD > 50% (*n* = 241)	No CAD > 50% (*n* = 73)	p
Age, median (IQR)	66 (17)	67 (15)	63 (16)	0.005
Male Gender (%)	229 (73%) (57%)	186 (77%)	43 (59%)	0.002
Hypertension (%)	227 (72%)	183 (76%)	44 (60%)	0.008
Family history of CAD (%)	93 (30%)	72 (30%)	21 (29%)	0.855
Smoking (%)	120 (38%)	99 (41%)	21(29%)	0.057
Hypercolesterolemia (%)	188 (60%)	152 (63%)	36 (49%)	0.035
Diabetes mellitus (%)	71 (23%)	63 (26%)	8 (11%)	0.006
Prior Miocardial infarction	73 (23%)	62 (26%)	11 (15%)	0.059
Coronary stenosis >50%	241 (77%)	241 (100%)	0	–
At least one stenosis more than 70%	222 (71%)	222 (92%)	0	–
Any reversible WM abnormality	206 (66%)	172 (71%)	35 (48%)	0.0002
More than mild reversible WM abnormality (>0.06)	164 (52%)	146 (61%)	18 (25%)	<0.0001
Reversible myocardial perfusion abnormality	136/176 (77%)^a^	105/130 (81%)^a^	31/46 (67%)^a^	0.063
CFR-LAD < 2	110/208 (53%)^a^	87/151 (58%)^a^	23/57 (40%)^a^	0.026
Normal WM + normal or unavailable myocardial perfusion and CFR-LAD	59 (19%)	44 (18%)	15 (20%)	0.66

*CAD* coronary artery disease, *IQR* interquartile range, *WM* wall motion, *CFR-LAD* coronary flow reserve of the left anterior descending coronary artery

^a^Myocardial perfusion and CFR-LAD percentages are related to the total number of patients in whom such variables could actually be technically measured

The presence of any degree of reversible WM abnormality during cSE was more frequent in the CAD (71%) vs no CAD (48%) groups (*p* < 0.001) but also present in almost half of patients with no stenosis < 50% at iCA, while when considering positive for ischemia only more-than-mild reversible WM abnormalities (WMSI delta > 0.06) such false positive rate of WM abnormalities fell to 29%. The only other cSE imaging variable differing between the 2 groups was a reduced CFR-LAD < 2 (best cutoff calculated from ROC curve in this iCA group was 1.96 and we decided to use the literature standard 2.0), which was significantly more frequent in the CAD group vs no CAD group (58% vs 40%, *p* = 0.026).

**Fig. 6:**
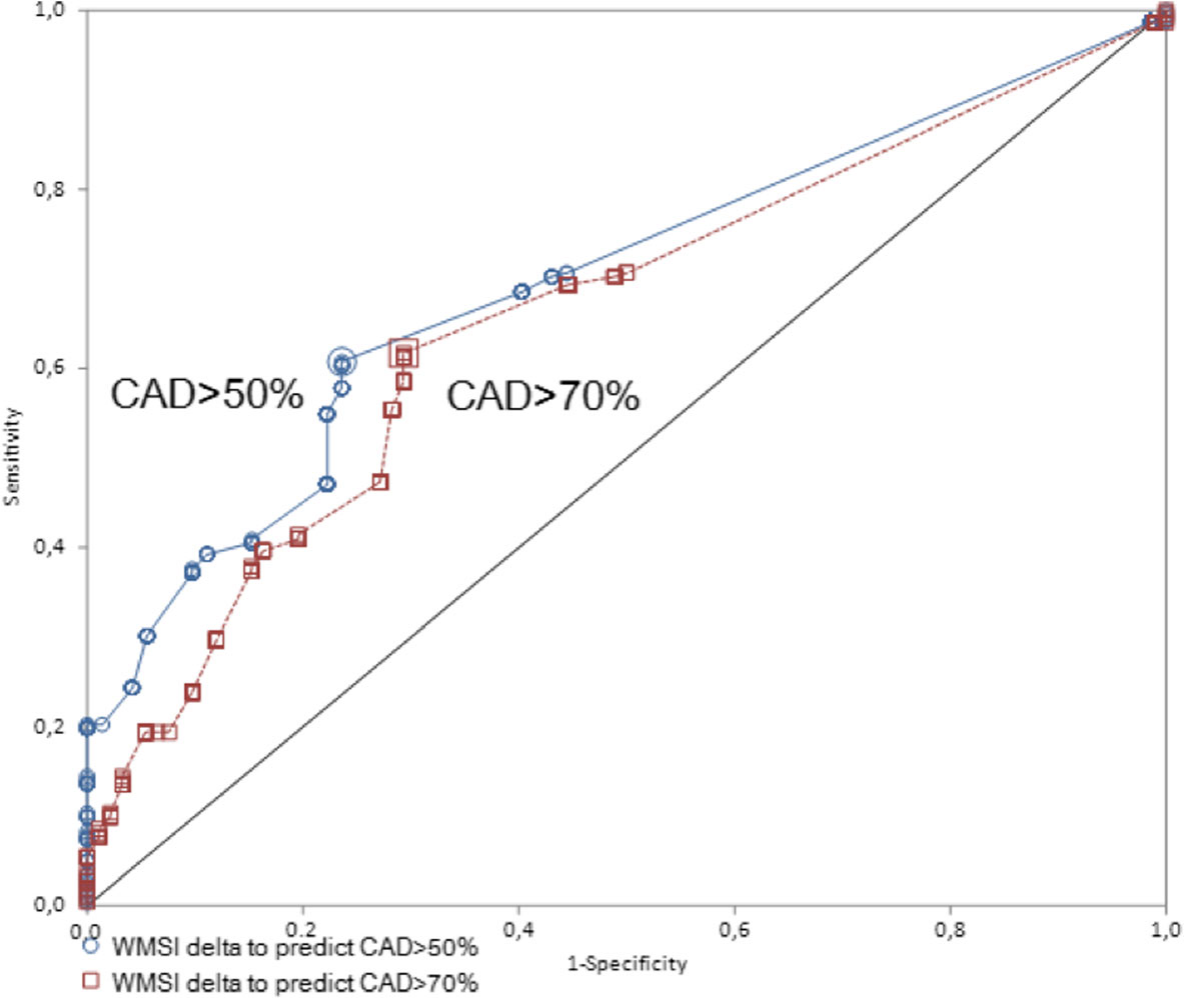
Delta wall motion score index to predict either CAD > 50% or CAD > 70%. CAD = coronary artery disease

Figure 6 shows the ROC curves for reversible WM abnormalities based on WMSI delta to predict CAD, for CAD > 50% demonstrating an AUC = 0.696 (95% CI 0.636 to 0.756), a negative predictive value of 63,3% (95% CI 55% to 71%) and a positive predictive value of 90% (95% CI 84% to 94%), and for CAD > 70% an AUC = 0.658, 95% CI 0.596 to 0.720), but a negative predictive value of 57% (95% CI 48% to 65%) with a positive predictive value of 83.5% (95% CI 77% to 89%).

**Fig. 7:**
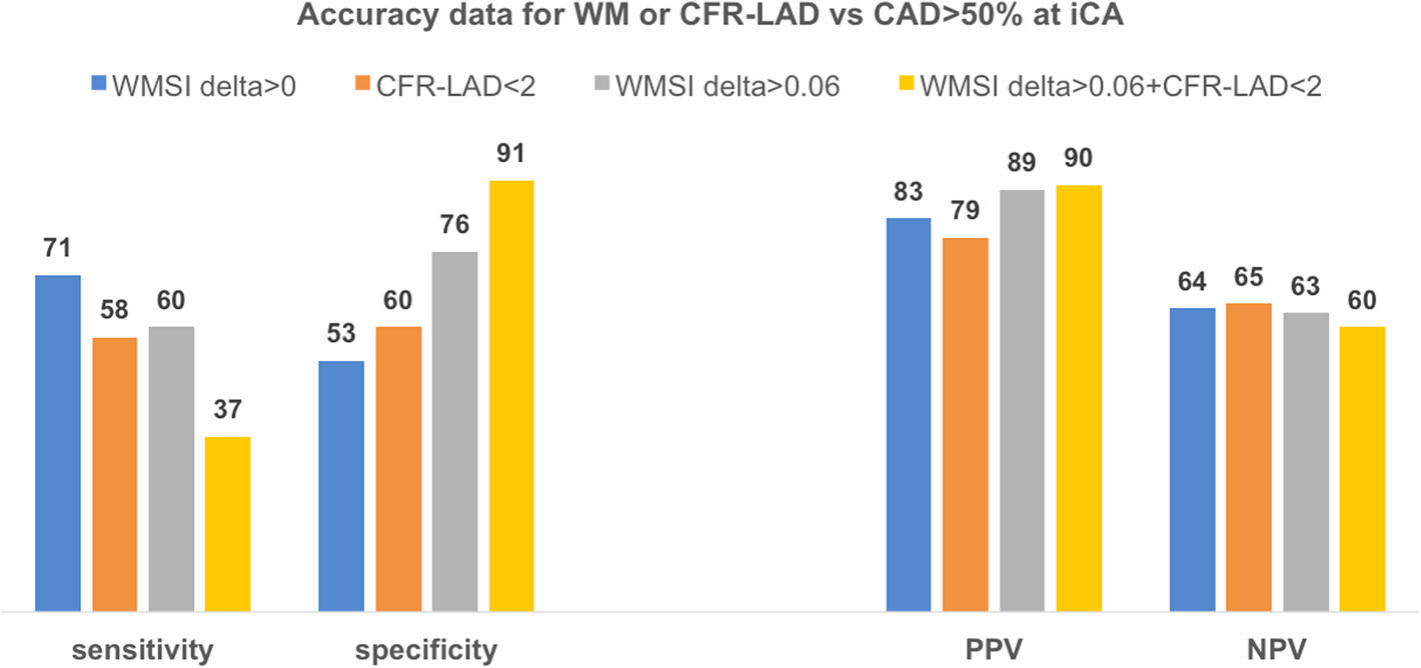
Accuracy data to predict CAD >50% at iCA, when WMSI or CFR-LAD were assessed as predictors. CAD = coronary artery disease, PPV = positive predictive value, NPP = negative predictive value, WM = wall motion, WMSI = wall motion score index, CFR-LAD = coronary flow reserve of the left anterior descending coronary artery It should be noted that while wall motion data were collected in 100% of patients (314/314), CFR-LAD measurement was available only for 209/314 patients (66%), due to lower technical feasibility. CFR-LAD accuracy data are calculated using available data and excluding patients in whom CFR-LAD was not measurable.

Full accuracy data in this iCA group are shown in Fig. 7, which highlights the high positive predictive value of cSE data, in particular of a more than mild reversible WM abnormality (WMSI delta >0.06) to predict CAD; CFR-LAD appeared less useful and also the combination of the two parameters did not increase significantly the predictive value of simple WMSI delta >0.06. On the other hand, all tested variables demonstrated a low negative predictive value for CAD.

**Fig. 8:**
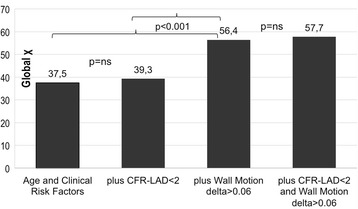
Incremental usefulness based on model chi square of adding imaging data over baseline demographics and clinical variables to predict CAD > 50%. CAD = coronary artery disease. CFR-LAD = coronary flow reserve of the left anterior descending coronary artery

Figure 8 graphically demonstrates that WMSI data (delta >0.06) add to baseline demographics and risk factors for the prediction of CAD >50% (chi square increases from 37.5 to 56.4, *p* < 0.001) while adding CFR-LAD data did not (chi square increase from 37.5 to 39.3, p = ns).

## Discussion

Our data confirm the common sense that once a functional imaging test for suspect CAD is performed, only patients showing more than mild degree of ischemia, in this case reversible WM abnormality, should be indicated iCA, by so doing possibly decreasing to trivial or “one-digit” percentage the subsequent “normal” findings of non-obstructive CAD at iCA.

This was never specifically confirmed for stress-echocardiography and it is not a trivial issue, since many (if not most) positive stress-echocardiograms performed in the low CAD prevalence population, which nowadays undergoes functional testing, demonstrate limited WMSI delta; the physician is consequently left with a clinical dilemma regarding what to do next in these cases.

Patients with equivocal results or only mildly positive WM behavior are not immune to CAD, but they may be rather indicated non-invasive CTA; further, coronary calcium score could be used first in this case, as a gatekeeper, to decide whether to proceed or not to full contrast CTA, based on an Agatston score threshold of 100 (an easy cutoff to memorize), since a lower score in our cohort demonstrated a very high (99%) negative predictive value for CAD > 70%, which would not justify routinely proceeding to CTA. Non-contrast CT calcium scoring is cheaper and biologically safer than CTA (even more if compared with iCA) for both being non-invasive and implying only trivial radiation.

**Fig. 9:**
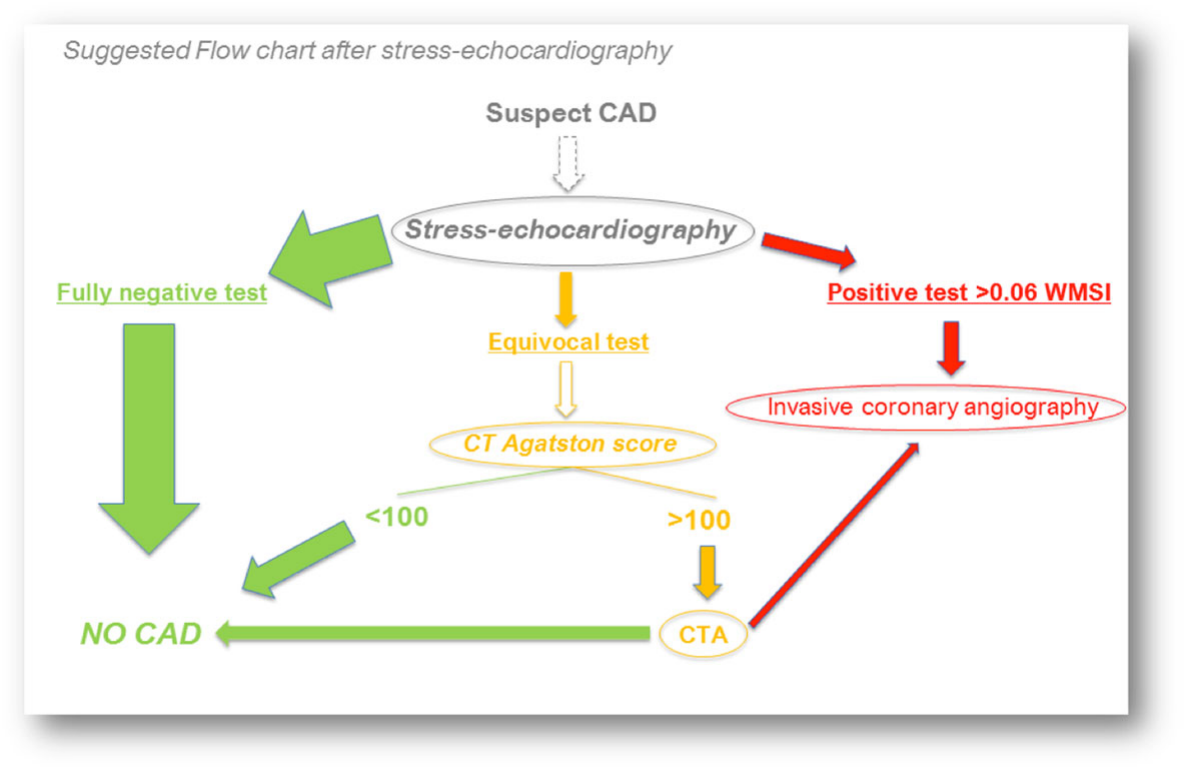
Diagnostic flow-chart suggesting a rational and evidence-based use of CT, CTA and invasive coronary angiography after stress-echocardiography. The width of the arrows graphically represents the percentage or number of patients who follow one or the other way in the flow-chart, depending on tests results. CAD = coronary artery disease. CT = computed tomography. CTA = computed tomography angiography, WMSI = wall motion score index

The implementation of this hypothetical flow-chart (Fig. 9) derived from our dataset to optimize the diagnostic process after cSE would possibly reduce the number of equivocal cSE finally proceeding to iCA, but also to full CTA, simply using a 0–100 Agatston score to skip subsequent CTA, and still identifying the same overall number of patients with CAD. This strategy is retrospectively derived by our data, and should definitely be confirmed in a prospective validation cohort.

CFR-LAD less than 1.7 in cSE equivocal tests also demonstrated a high negative predictive value, almost as good as the CT Agatston score, but suboptimal feasibility of this measurement may be perceived as an issue for less experienced operators.

Although most patients with an equivocal cSE were finally reassured by CTA regarding the absence of anatomically severely obstructive CAD, this group with equivocal cSE tests hides a non-trivial percentage of patients with CAD who should not be overlooked or simply reassured.

### Why not “go functional” if accuracy could be similar?

While the Recent NICE guidelines [2] suggest that CTA should be preferred over functional imaging stress-testing in the suspicion of CAD, and we mostly agree due to the limited diagnostic accuracy of both ECG or imaging stress-tests when used as gatekeepers to iCA [9, 10], CTA could instead more realistically play the role of the missing link between functional tests and iCA. This would result particularly effective for the “grey zone” of patients with equivocal tests showing no or mildly abnormal WM behavior, who could be more confidently indicated iCA, maximizing the percentage who are finally diagnosed with obstructive CAD, and minimizing false-positive cSE undergoing iCA.

Starting from a functional test, instead of an anatomical one, has the main advantage that semi-quantitative inducible ischemia data are still today the mainstay (together with invasive coronary fractional flow measurements) to indicate subsequent coronary revascularization in stable CAD. Consequently, diagnostic, prognostic and therapeutic indications may all be assessed within a single test, in the specific case of cSE with a very low biological and economic cost, compared with a “CTA-for-all” strategy. CTA also has non-zero false positive rate compared with iCA, as it is apparently also confirmed in our study, which would be amplified if used indiscriminately.

### Limitations

This is a retrospective and single-center study and the strategy suggested needs to be further tested in a prospective and multicenter validation cohort.

Contrast stress-echocardiograms were routinely performed by 4 different operators and cSE interpretation is to some degree subjective, although it has been demonstrated that using contrast for endocardial border enhancement, as it was in our study in 100% of studies, the variability of interpretation is very significantly reduced [11, 12].

Myocardial perfusion and CFR-LAD data were not available for all patients, mostly due to the multiple operators involved in the clinical routine of our echolab, which is the setting of this study, not all being specifically trained or skilled in myocardial perfusion imaging and CFR-LAD, but WM remains the mainstay and the most widely used marker during stress-echocardiography to indicate iCA, and our diagnostic strategy in fact focused on WM behavior. The finding of myocardial perfusion data resulting almost useless to predict the presence of obstructive CAD (the prevalence of reversible perfusion defects was not significantly different between CAD and no CAD groups) may derive from cSE being performed by several different operators after 2012 in our lab, only rarely skilled in this complicated imaging method and interpretation, differently from prior studies published from our lab, when myocardial perfusion assessments were conducted and interpreted mostly by a single and experienced operator.

## Conclusions

Our data suggest that iCA should be indicated only for more than mild reversible ischemia by WM assessment at cSE, due to the very high positive predictive value of this variable for CAD, while mildly positive tests may rather be shifted to non-invasive CTA. Further, coronary calcium score could be assessed, as a gatekeeper, to proceed only in case of an Agatston score > 100 to CTA, since a lower score in our cohort demonstrated such a high negative predictive value for CAD that it would not justify proceeding to CTA. A significant percentage of patients with mildly positive cSE today usually sent to iCA would be more efficiently managed shifting them to non-contrast CT calcium scoring first, which is cheap and biologically safer for both being non-invasive and implying only trivial radiation.

## Abbreviations

AUC: Area under the curve
CAD: Coronary artery disease
CFR-LAD: Coronary flow reserve of the left anterior descending coronary artery
cSE: Contrast stress-echocardiography
CT: Computed tomography
CTA: CT angiography
ECG: Electrocardiogram
iCA: Invasive coronary angiography
ROC: Receiver operating characteristic
WM: Wall motion
WMSI: Wall motion score index
